# Time-Varying Kelvin Wake Model and Microwave Velocity Observation

**DOI:** 10.3390/s20061575

**Published:** 2020-03-12

**Authors:** Jie Niu, Xingdong Liang, Xin Zhang

**Affiliations:** 1 National Key Lab of Microwave Imaging Technology, Aerospace Information Research Institute, Chinese Academy of Sciences, Beijing 100190, China; niujie15@mails.ucas.ac.cn (J.N.); xdliang@mail.ie.ac.cn (X.L.); 2 School of Electronics, Electrical and Communication Engineering, University of Chinese Academy of Sciences, Beijing 100049, China

**Keywords:** Kelvin wake, time-varying model, MIMO, millimeter wave radar, Doppler velocity observation

## Abstract

In the synthetic aperture radar (SAR) imaging of ship-induced wakes, it is difficult to obtain the Doppler velocity of a Kelvin wake due to the lack of time-varying wake models and suitable radar equipment. The conventional Kelvin wake investigation based on the static Kelvin wake model failed to reflect time-varying characteristics, which are significant in the application of the Kelvin wake model. Therefore, a time-varying Kelvin wake model with consideration of geometric time-varying characteristics and the hydrodynamic equation is proposed in this paper, which reflects the wake’s time-varying change lacking in the conventional Kelvin wake investigation. The Doppler velocity measurement, measured by a specially designed radar, can be exploited to verify the time-varying model by the comparison of velocity fields. Ground-based multi-input multi-output (MIMO) millimeter wave radar imaging through the simultaneous switching of transceiver channels was used to obtain the Doppler velocity for the first time. Finally, promising results have been achieved, which are in good agreement with our proposed model in consideration of the experimental scene. The proposed time-varying model and radar equipment provide velocity measurements for the Kelvin wake observation, which contains huge application potential.

## 1. Introduction

Ship-induced Kelvin wakes have been proved to be more visible than the ships with low radar cross section (RCS) in synthetic aperture radar (SAR) images, which is of great significance to sea surface target detection and identification as well as the parameter inversions. The model of Kelvin wakes was firstly proposed by Lord Kelvin, which combines the experiment observation with fluid dynamic theory. With the help of the model, scholars have done lots of researches on Kelvin wakes with different observation methods. 

Microwave radar has been proven to be useful in observing the Kelvin wakes. The feature of Kelvin wakes in SAR images and the electromagnetic scattering characteristics from the Kelvin wakes are widely discussed [1,2,3,4,5,6,7,8,9,10,11,12,13]. Based on the Bragg mechanism of the sea surface, Tunaley et al. [1] researched the SAR imaging of ship wakes in L-band and analyzed its validity through a comparison with the Sea SAT SAR imagery. Oumansour et al. [2] investigated the SAR imaging of ship wakes in an X- and L-band utilizing the small perturbation scattering model. Shemer et al. [3] described a mathematical model for imaging the ship wakes with the help of the interferometric SAR (INSAR) technique. Henning et al. [4] gave an explanation of the principle of SAR imaging of Kelvin arms and discussed the simulated results with experiments. Arnold Bos et al. [7] developed a bistatic, polarimetric radar simulator for estimating pseudo-raw radar echoes of ship wakes that can be further processed for bistatic SAR (BiSAR) imaging. The detectability of the boundaries of Kelvin wake in SAR images is studied with the application of discrete Radon transform by Zilman et al. [9]. Meng et al. [10] analyzed the electromagnetic scattering characteristics of Kelvin wakes on the rough sea surface with the help of the Integral Equation Model (IEM). Rui et al. [11] utilized the second-order small-slope approximation to investigate electromagnetic scattering from the sea surface with Kelvin wakes. Recently, Nan et al. [12] studied the method for detecting and locating a Kelvin wake in the rough sea surface by partitioning the sea surfaces with feature selective validation method.

Most of the investigation is based on the static model of Kelvin wakes without the factor of time-varying which belongs to the traditional model by Lord Kelvin from a long time ago. Previous simulation and experiments of Kelvin wakes are based on the static model as well, which is difficult to reflect time-varying characteristics. Most of imaging radars in previous experiments of the Kelvin wake also focused on static observations without the ability to observe time-varying characteristics, such as Doppler velocity. For further application of the Kelvin wake, one would like to have a sophisticated model like this, which is applicable in the wakes’ Doppler velocity with respect to the time-varying and to observe the time-varying Kelvin wake by a suitable imaging radar with the function of Doppler velocity measurement. In this paper, a time-varying Kelvin wake model that basically meets the requirement is firstly presented. The formulation of the time-varying Kelvin wake model based on the original Kelvin wake model is investigated and the accuracy of the model is proved by the velocity observation experiment in this paper. With the dynamic observation of the Kelvin wake, the ability to exploit the information of the Kelvin wake will be promoted. For example, with the help of dynamic observationson the Kelvin wake, it can be possible to distinguish a speedboat’s wake from a cruise’s wake, which provides a new method to detect pirates.

The rest of this paper is structured as follows. In Section 2, the formulation of the static model of a ship-generated Kelvin wake and time-varying model with simulation is introduced. In Section 3, a ground-based multi-input multi-output (MIMO) SAR with the ability to measure Doppler velocity and field experiment is briefly described. In Section 4, data processing based on an MIMO SAR are proposed to observe the Kelvin wake. In Section 5, results analysis compared with time-varying model are introduced. Section 6 concludes this article.

## 2. Hydrodynamic time-varying Model of Kelvin Wakes

**Figure 1:**
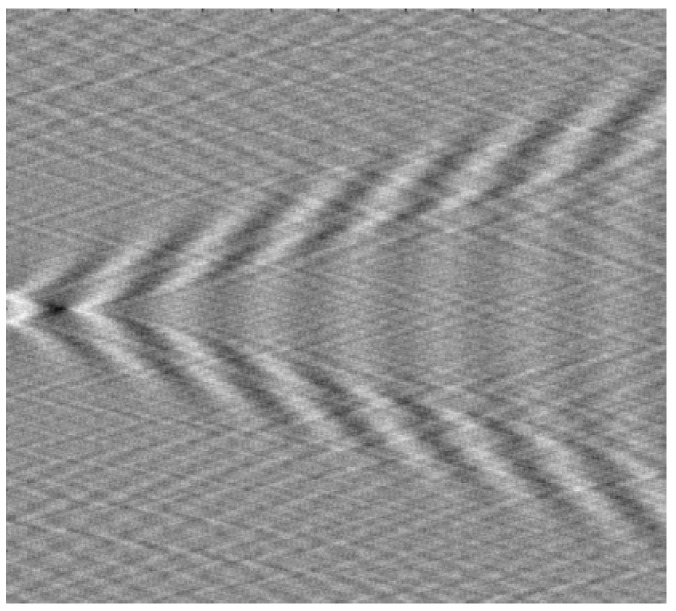
Kelvin wake pattern of aship velocity of 5 m/s.

Kelvin wake is one of the infragravity waves shown in Figure 1. The formulation of a ship-generated Kelvin wake was firstly concluded mathematically by Lord Kelvin, which treats the moving ship as an ideal point disturbance. The formulation highlights that the wave patterns of a Kelvin wake mainly comprise transverse waves and divergent waves. The waves mentioned above form a cusp wave with the help of interference. The cusp wave is located in the area where the wake surface is the most undulating. Due to the short wavelength of the cusp wave, each wavefront is nearly impossible to be independently distinguished, which appears as a bright line called a Kelvin arm. According to the computation and experiments, the angle of the Kelvin arm is $\pm 19.5^{\circ }$.

Based on Lord Kelvin’s theory, the wave elevation propagating at different angle sgenerated by a ship moving with speed $U_{s}$ in the x direction can be written as follows [4]:

(1) ςk(x,y)=Re∫−π/2π/2F(θ)exp[−ikksec2θ(xcosθ+ysinθ)]dθ

where kk=gUs2tanh(kH) represents infinite water depth and kk=gUs2 represents finite water depth, g is the acceleration due to gravity, $U_{s}^{}$ is the velocity of the ship, $k_{k}\sec^{2}\theta \left(x\cos\theta +y\sin\theta \right)$ is the phase modulation function, $k_{k}\sec^{2}\theta$ is the wave number of the waves travelling at angle θ, and F(θ) is the free spectrum that depicts the ship’s characteristics, which can be written as follows [12]:

(2) $$F\left(\theta \right)=\frac{2k_{k}}{\pi }\sec^{3}\theta \iint \frac{\partial Z\left(x,z\right)}{\partial x}\exp\left[k_{k}\sec^{2}\theta \left(ix\cos\theta +z\right)\right]dxdz$$

where Z=Z(x,z) is the hull equation of the ship. If we consider a simple hull shape with parabolic waterlines, and if it is a wall-sided ship with draft depth d, then:

(3) $$Z\left(x,z\right)=\{\begin{array}{ll} b\left(1-x^{2}/l^{2}\right), & -d\leq z\leq 0,-l\leq x\leq l\\ 0, & z<-d \end{array}$$

where b is the half-beam and l is the half-length of the ship. 

The active microwave remote sensing radar, Synthetic Aperture Radar (SAR), is widely exploited in Kelvin wake observation. The microwave imaging mechanism of the Kelvin wake can be simplified to a two-step process: First, the movement of the ship produces a Kelvin wake, which leads to a modulation of the wave height. Then, the changed wave height due to the Kelvin wake gives rise to the hydrodynamic modulation of the surface roughness, which makes the RCS of the wake’s area different from the area without a Kelvin wake. The time-varying difference of RCS due to the Kelvin wake can be detected by radars. However, traditional SAR images are focused on still scenes. The SAR images of dynamic scenes are blurry. Thus, the time-varying model of Kelvin wake needs to be derived to improve the SAR imaging of Kelvin’s wake.

With the consideration of the influence of time variations on Kelvin’s wake, a time-varying model of the Kelvin wake is proposed based on the original Kelvin wake formulation, geometric time-varying characteristics, and the hydrodynamic equation. The geometric time-varying characteristics is reflected in the displacement of the target in the direction of motion over time. The hydrodynamic equation is reflected in the propagation attenuation. The basic form of the hydrodynamic equation is as follows:

(4) $$\frac{\partial v}{\partial t}+\left(v\cdot \nabla \right)v=-\frac{1}{\rho }\nabla P+g$$

where v is the velocity of fluid flow, ρ is the fluid density, P is the fluid pressure, and g is acceleration due to gravity. When the detailed expression is applied in the Kelvin wake model, the solution about $J_{0}\left(kr\right)$ should be attached to the Kelvin wake form as the propagation attenuation. Assuming that the ship is in a uniform rectilinear motion, the time-varying model of a Kelvin wake is derived as:

(5) ςk(x,y,t)=Re∫−π/2π/2J0(kktan(19.5∘)Ust)A(θ)exp[−ikksec2θ((x+Ust)cosθ+ysinθ)]dθ

where $J_{0}$ is the first order Bessel function derived by hydrodynamic equation. It can be seen from the time-varying model that the formulation accounts not only for the wave propagation with time but for the geometrical feature of the wake.

**Figure 2:**
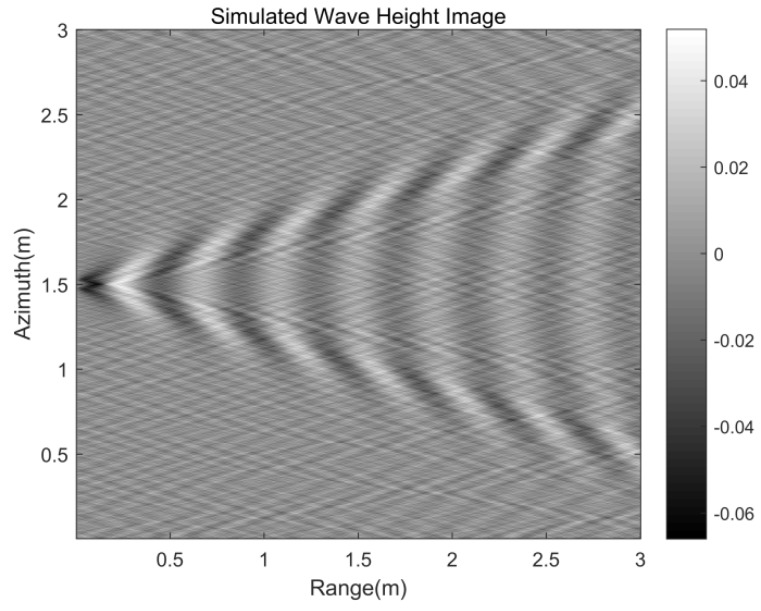
Kelvin wake wave height image.

**Figure 3:**
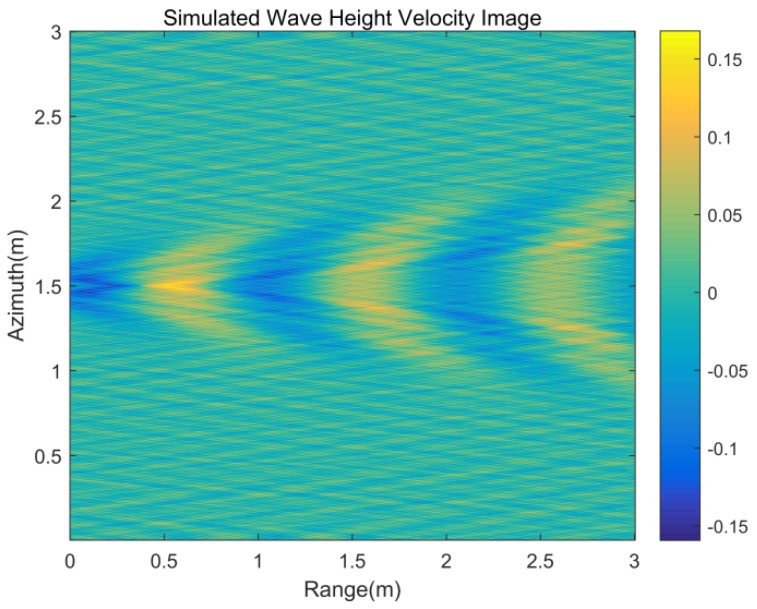
Kelvin wake wave height velocity image.

Table 1 presents parameters of the target to simulate the Kelvin wake, which are basically consistent with the actual experimental target parameters. Figure 2 is the wave height of the static Kelvin wake model under parameters from Table 1. Figure 3 is the velocity of the time-varying model under the same parameters.

## 3. Radar and Field Experiment

**Figure 4:**
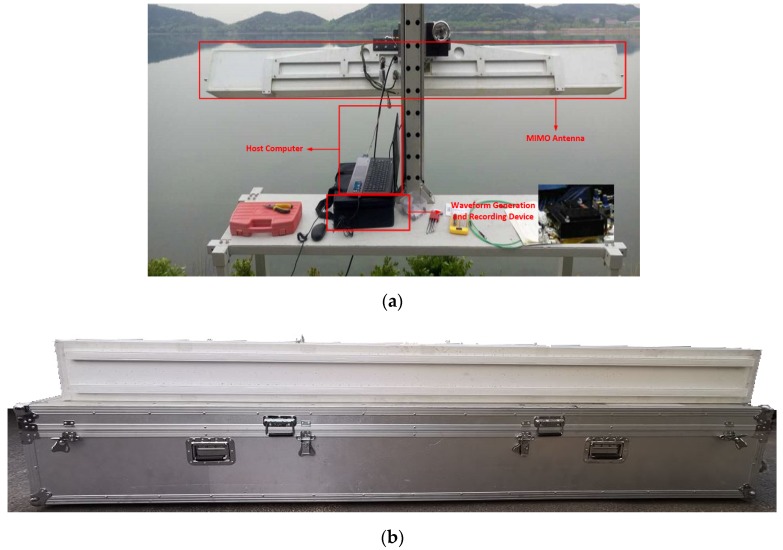
Ground-based multi-input multi-output synthetic aperture radar (MIMO SAR). (**a**) The structure of the radar; (**b**) the MIMO antenna.

A ground-based MIMO SARat Ka band (34.6 GHz) is exploited as the scattering and velocity observation facility, which consists of the host computer, waveform generation, and a recording device and MIMO antenna, as shown in Figure 4a. The equipment mentioned in this paper is the first millimeter waveradar to achieve imaging through simultaneous switching of transceiver channels. The ground-based MIMO SAR owns higher range and azimuth resolution than the existing ground-based imaging radar mentioned in published articles [14,15,16]. A linear frequency modulation signal is generated by the waveform generation and is saved by recording device. The antenna switching sequence that controls the antenna array is sent by the host computer. The frequency of the antenna switching is determined by the pulse repetition frequency (PRF). On the antenna part, a linear array of transmit antennas with 120 transmit elements and a linear array of receive antennas with 120 receive elements are correspondingly addressed, as shown in Figure 4b, which provides different antenna switching modes. The length of the whole antenna is 280mm and the height is 30 mm. Both transmit antennas and receive antennas are in vertical polarization. Different antenna switching modes can be exploited in a ground-based MIMO SAR, which makes it possible to emulate SAR images with different platform velocities and azimuth sampling densities. One of the most common switching modes with a high resolution will be explained in the next section.

The ground-based MIMO SAR can also mimic a multichannel SAR (MSAR) to measure velocities by rapidly and repeatedly scanning across the MIMO antenna as a specific role. The spacing between the array elements is $d_{MIMO-SAR}=0.02m$. Each image generated by the ground-based MIMO SAR is high-resolution and real-time, with the shortest time interval being 1 ms. The range resolution is 0.125 m and the azimuth is 0.02 m, which performs better than other similar devices [16]. 

**Figure 5:**
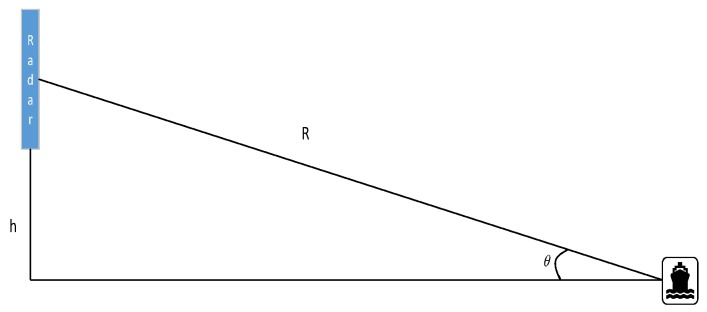
Experimental geometric observation model.

A closed lake was chosen as the experiment area where the current influence is relatively small. The radar was deployed on the shore 1.9 m above the mean lake level. The geometrical diagram of the radar and target is shown in Figure 5. Simultaneously, the anemometer is set on a tower 10 m above the mean lake level to measure the wind velocity. During the experiment, the target motion direction is parallel to the radar array direction as the radar azimuth direction. The target with the ability to generate the Kelvin wake shown in Figure 2 has a velocity of 0.8m/s. Based on the Doppler velocity measurement of MSAR, the ground-based MIMO SAR owns the radial velocity acquisition capability. In order to obtain the velocity of the Kelvin wake compared with the time-varying model, the radial velocity simulation of Kelvin wake is necessary. The radial observation velocity of the Kelvin wake $v_{R}$ consists of the wave height velocity component $v_{h}$ and the wake velocity perpendicular to the motion direction component $v_{r}$. Therefore, the radial velocity can be expressed as follows:

(6) $$v_{R}=v_{h}+v_{r}$$

**Figure 6:**
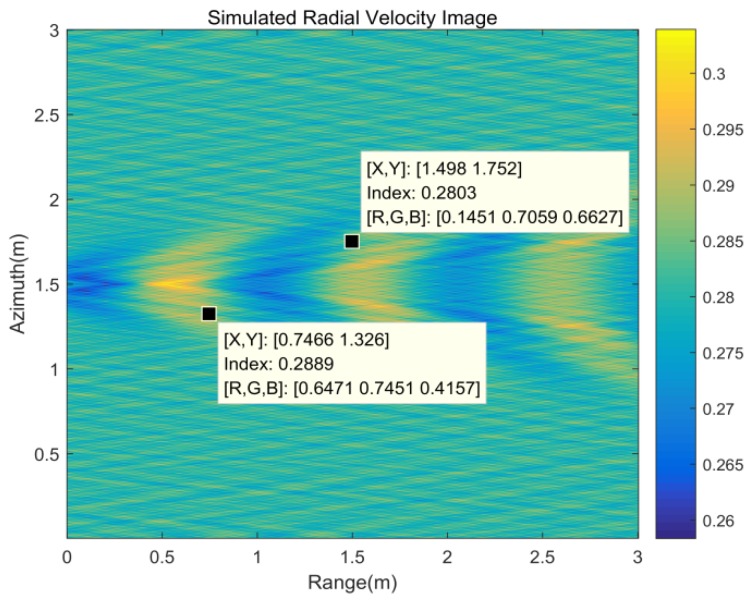
Kelvin wake wave radial velocity image.

Combined with the target parameters and experiment geometric, the Kelvin wake radial velocity image generated by the target motion can be simulated under the time-varying model. Figure 6 shows the simulation of the radial velocity of the Kelvin wake. From the simulation result (Figure 6), the radial velocity of Kelvin arms ranges from 0.2803 to 0.2889 m/s and the radial velocity of the whole Kelvin wake ranges from 0.2584 to 0.3039 m/s. 

## 4. Data Processing

### 4.1. Radar Imaging

**Figure 7:**
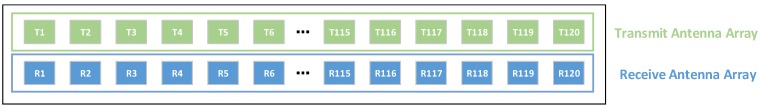
Schematic diagram of the transceiver antenna array.

As described in Section 3, the ground-based MIMO SAR obtains a high-range resolution using a linear frequency modulation (LFM) signal and a high azimuth resolution by mimicking the synthetic aperture. In particular, variant antenna switching modes generate variant azimuth sampling densities. The schematic diagram of the transceiver antenna array is shown in Figure 7. An optimized switch mode called a cross-order switch mode is configured to obtain better azimuth imaging result though the reduction of phase center sampling spacing, which meets the PRF requirements of SAR imaging. During the first pulse repetition interval (PRI), only the transmit antenna T1 and receive antenna R1 in Figure 6 are connected to the transceiver. During the second PRI, the switches are reconfigured such that the T1 and R2 are addressed instead of T2 and R2 in the sequential switching mode. During the next PRI, the switches are reconfigured such that the T2 and R2 are addressed. This is repeated until the last transmit and receive antenna are connected. In this mode, the azimuth resolution of image is 0.01m and the time interval between each image is in a period of approximately 2 ms, which is well within the decorrelation time for microwave backscatter at Ka band [17,18]. The short interval time means that the scattering of the water surface can be reflected more realistically.

Ground-based MIMO SAR imaging processing is similar to SAR, and the transmit LFM signal is written as follows:

(7) $$s_{1}\left(t\right)=rect\left(\frac{t}{T_{p}}\right)A\sin\left(\pi K_{r}t^{2}+2\pi f_{c}t+\varphi_{0}\right)$$

where $T_{p}$ is pulse width, A is the amplitude of the transmit signal, $K_{r}$ is frequency modulation rate, $f_{c}$ is center frequency, and $\varphi_{0}$ is initial phase.

Assuming that the target is at a position with a distance of R, the delay generated by the distance is as follows:

(8) $$\tau =\frac{2R}{c}$$

Thus, the receiving signal can be written as follows:

(9) $$s_{2}\left(t\right)=rect\left(\frac{t-\tau }{T_{p}}\right)B\sin\left[\pi K_{r}{\left(t-\tau \right)}^{2}+2\pi f_{c}\left(t-\tau \right)+\varphi \right]$$

where B is the amplitude of the transmit signal determined by a radar two-way antenna pattern and target backscattering characteristics.

Under the LFM signal process, the transmitted signal is used as a reference signal, which is mixed with the receive signal. The mixed signal is as follows:

(10) $$s_{3}\left(t\right)=rect\left(\frac{t-2R/c}{T_{p}}\right)C\cos\left[\frac{4\pi R}{c}\left(K_{r}t+f_{c}-\frac{K_{r}R}{c}\right)+\varphi -\varphi_{0}\right]$$

where C is the amplitude of the mixed signal related to A,B, and the system parameters. The mixed signal is a single frequency cosine signal with the frequency of:

(11) $$f=\frac{2K_{r}R}{c}$$

By performing a Fourier transform on the mixed output signal, the range pulse compression can be completed. The azimuth pulse compression is then performed according to the array switching velocity to complete the imaging process.

### 4.2. Velocity Imaging Process

The ground-based MIMO SAR can not only acquire a single radar image through fast imaging scanning but can also perform Doppler processing along the time dimension on the acquired time-related multiple radar images to obtain a velocity image.

From the basic introduction of the ground-based MIMO SAR in Section 3 the scanning time for one image under the cross-order switch mode is as follows:

(12) $$t_{scan}=2N\cdot PRT$$

where N is the number of transmit/receive elements in the ground-based MIMO SAR array. With the utilization of a plurality of continuous scan and time-related images, a set of images with a time interval of $t_{scan}$ is contrasted. The ground-based MIMO SAR Doppler velocity process is similar with the velocity SAR (VSAR) Doppler velocity process. The effective spacing is computed as the product of the interval between images and the rate at which the scan progresses across the following array [19,20]:

(13) $$d_{VSAR}=\frac{d_{MIMO}}{2PRT}t_{scan}$$

where $d_{MIMO}$ is the spacing of the adjacent transmit/receive elements whose value is 0.02 m.

The unambiguous Doppler velocity and velocity resolution for the ground-based MIMO SAR can be expressed in the same form as for the following VSAR:

(14) $$v_{\max}=\frac{v_{VSAR}\lambda }{2d_{VSAR}}$$

(15) $$\Delta v=\frac{v_{VSAR}\lambda }{Md_{VSAR}}$$

where vVSAR=dMIMO2PRT, M is the number of chosen time-related images exploited in the Doppler velocity process.

With the above derivation, the unambiguous Doppler velocity and velocity resolution can be computed combined with MIMO ground-based SAR specific parameters. The unambiguous Doppler velocity of the ground-based MIMO SAR is 1.128 m/s and the velocity resolution is 0.141 m/s. According to the velocity simulation with the time-varying model of the Kelvin wake, the ground-based MIMO SAR has the proper velocity observation ability to observe the velocity of the Kelvin wake.

## 5. Result Analysis

The experiment based on the ground-based MIMO SAR was carried out in a closed lake to avoid the influence of the current. An Unmanned Underwater Vehicle (UUV) was exploited as the target to generate the Kelvin wake. 

**Figure 8:**
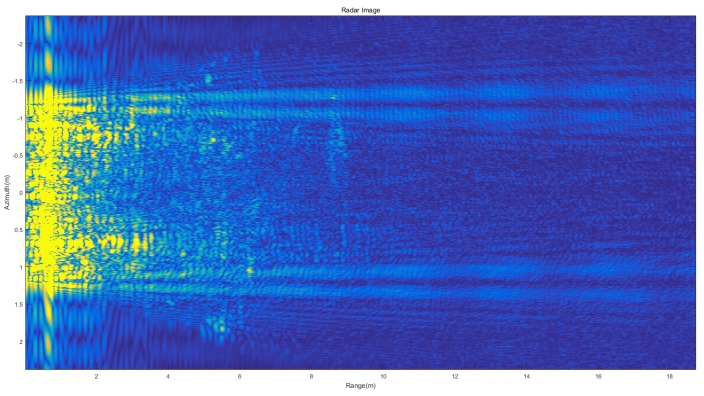
Static Kelvin wake radar image.

**Figure 9:**
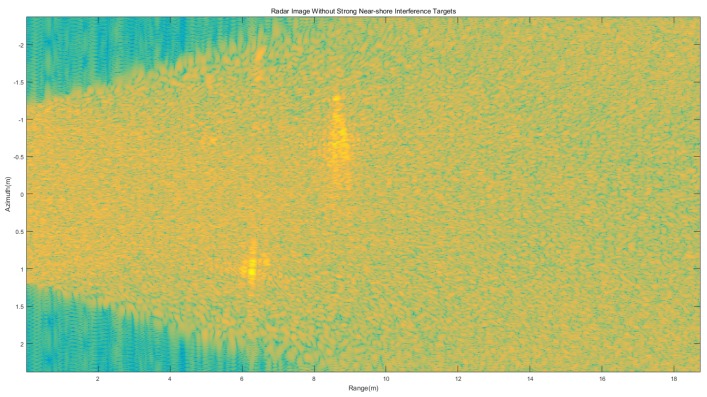
Static Kelvin wake radar image without strong near-shore interference targets.

The target moved along the array antenna direction in the experiment with the velocity of 0.8m/s, which was the same as the simulation velocity. The radar image of the static Kelvin wake is shown in Figure 8. With the simple Doppler process, the strong near-shore interference targets with the velocity of 0 m/s were removed, as shown in Figure 9.

The radial velocity measured by the ground-based MIMO SAR consists of the following parts [16]:

(16) $$v_{r}=v_{wake}+v_{Bragg}+v_{current}$$

where $v_{wake}$ is the velocity generated by the Kelvin wake, $v_{Bragg}$ is the velocity generated by the Bragg wave phase velocity, and $v_{current}$ is the velocity generated by the current. 

**Figure 10:**
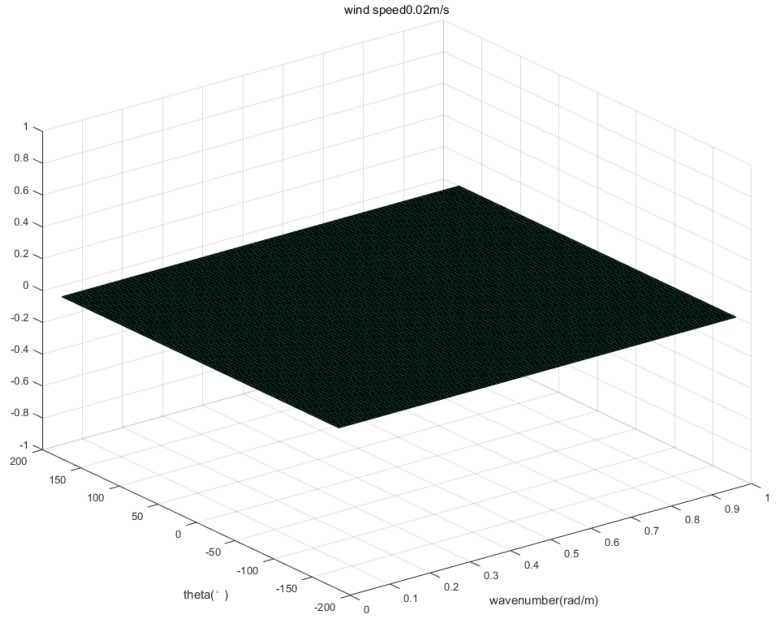
Two-dimensional wave spectrum with the measured wind speed.

**Figure 11:**
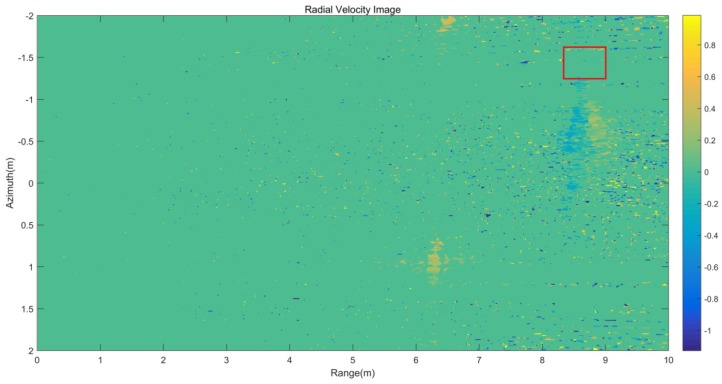
Kelvin wake radial velocity image.

The wind speed at 10m above the measured water surface is 0.02 m/s and the two-dimensional wave spectrum with the measured wind speed is shown in Figure 10. From the wave spectrum, the Bragg wavenumber component is close to 0 at this wind speed. Therefore, the velocity generated by the Bragg wave phase velocity $v_{Bragg}$ was negligible. As for the velocity generated by the current $v_{current}$, the area where the experiment was located is a closed area, and the current rate generated by the field is negligible. In order to verify the conclusion, the average velocity is calculated near the wake trace. The selected region is shown with the red area in Figure 11. After calculation, the average velocity of the region is 0.00021 m/s. From the above derivation, it can be seen that the measured radial velocity $v_{r}$ is the radial velocity component of the Kelvin wake $v_{wake}$.

**Figure 12:**
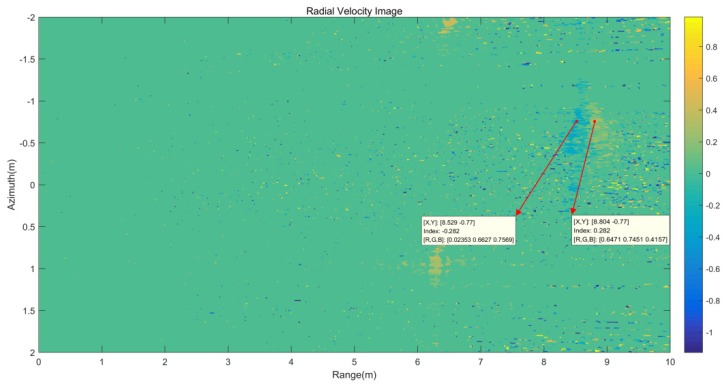
Kelvin wake radial velocity image.

According to the velocity image shown in Figure 12, the Kelvin wake velocity is bound by the wake centerline and the Kelvin wake arms velocities are −0.282 and 0.282 m/s. Positive Doppler velocities represent motion toward the radar. This result is consistent with the theoretical simulation results of the radial velocity in Section 2 with the time-varying model of the Kelvin wake, and the velocity is in the velocity range of the Kelvin arms from 0.2803 to 0.2889 m/s shown in Table 2.

## 6. Conclusions

In this paper, a time-varying model based on the original Kelvin wake model and the velocity simulation of the new model have been investigated in detail. The wave height and radial velocity images of the ship-generated Kelvin wake can be calculated with the time-varying model. The accuracy of the model is proved by a comparison between the simulated radial velocity image and the experimental Doppler velocity based on the ground-based MIMO SAR. 

With velocity observation of the Kelvin wake, the ability to exploit the information of the Kelvin wake will be promoted. It is possible for the velocity observation of the Kelvin wake to distinguish the wake with similar amplitudes in a radar image but with different radial velocities. It should be pointed out that the velocity observation based on a time-varying model of the Kelvin wake deserves further investigation to promote its application in a complicated scene, which goes beyond the scope of this paper.

## Figures and Tables

**Table 1:** Parameters of the Kelvin wake simulation target.

Parametric Name	Parametric Value
Half ship length	0.15 m
Half ship width	0.07 m
Side wall draft depth	0.02 m
Velocity	0.8 m/s

**Table 2:** Simulation and experimental radial velocities of the Kelvin wake arms.

Parametric Name	Parametric Value
Simulation radial velocity of the Kelvin wake arms	0.2803–0.2889 m/s
Experimental radial velocity of the Kelvin wake arms	0.282 m/s
